# Case Report: Advanced IgA nephropathy with psoriasis and a complex clinicopathologic course

**DOI:** 10.3389/fneph.2026.1932773

**Published:** 2026-09-08

**Authors:** Lerui Wang, Minxia Li, Boyang Xu, Dan Shu

**Affiliations:** 1 Beijing Tsinghua Changgung Hospital, School of Clinical Medicine, Tsinghua University, Beijing, China; 2 Department of Nephrology, Beijing Tsinghua Changgung Hospital, School of Clinical Medicine, Tsinghua University, Beijing, China; 3 Department of Dermatology, Beijing Tsinghua Changgung Hospital, School of Clinical Medicine, Tsinghua University, Beijing, China

**Keywords:** case report, deucravacitinib, IgA nephropathy, kidney biopsy, psoriasis, targeted-release budesonide, tyrosine kinase 2

## Abstract

Psoriasis and IgA nephropathy are immune-mediated disorders that may coexist, but the clinical meaning of this association remains uncertain. We report a 45-year-old man with psoriasis and progressive kidney dysfunction whose biopsy-proven IgA nephropathy presented with severe impairment of kidney function, heavy proteinuria, and mixed active and chronic histologic injury. At reassessment, serum creatinine was 463 μmol/L, estimated glomerular filtration rate was 12.1 mL/min/1.73 m², and pretreatment 24-hour urinary protein excretion ranged from 2.90 to 4.70 g. Kidney biopsy showed proliferative sclerosing IgA nephropathy, Oxford classification M1E1S1T2C0, with ischemic glomerular injury, microangiopathy, tubular atrophy, and interstitial fibrosis. Targeted-release budesonide and deucravacitinib were started together. Proteinuria fell to 0.62 g, serum creatinine decreased to 323 μmol/L, and a modest improvement in cutaneous disease activity was observed, with PASI decreasing from 2.4 to 1.6. After patient-initiated discontinuation of deucravacitinib, proteinuria increased and later declined after reintroduction. Following completion of the planned budesonide course, proteinuria continued to fall during deucravacitinib monotherapy, while kidney function deteriorated. This case is best interpreted as a clinicopathologic observation rather than proof of drug efficacy. Biopsy-defined activity and chronicity helped explain the discordant changes in proteinuria and kidney function, while the longitudinal course generated a testable but unproven hypothesis of a possible inflammatory connection between psoriasis and IgA nephropathy.

## Introduction

IgA nephropathy (IgAN) is a common primary glomerular disease and a cause of progressive chronic kidney disease. Current models emphasize mucosal immune dysregulation, production of galactose-deficient IgA1, immune complex formation, complement activation, and downstream glomerular injury (1, 2). Psoriasis is a chronic immune-mediated skin disease with systemic inflammatory features. Population-based and clinicopathologic studies have linked psoriasis with chronic kidney disease and glomerulonephritis, including IgAN, although the relationship is incompletely defined (3, 4).

This case was considered worth reporting because it provides more than documentation of coexisting psoriasis and IgAN. The patient had advanced biopsy-proven IgAN with active glomerular lesions and severe chronic structural damage in the setting of a more than 10-year history of severe, relapsing plaque psoriasis. The subsequent clinical course was also nonparallel: cutaneous disease activity, proteinuria, and kidney function did not evolve as a single outcome. By combining detailed renal pathology with longitudinal renal and dermatologic observations, this case illustrates how biopsy-defined activity and chronicity can frame prognosis and constrain interpretation of apparent treatment-associated changes. The observed phenotype is therefore clinicopathologic and hypothesis-generating; it is compatible with, but does not establish, a possible inflammatory connection between psoriasis and IgAN.

## Case description

### Patient information and previous interventions

A 45-year-old man was referred for nephrologic evaluation because of progressive kidney dysfunction and foamy urine. He had a more than 10-year history of severe, relapsing plaque psoriasis. The disease initially involved the lower extremities and later affected the face, trunk, and extremities. Earlier dermatologic examination documented erythematous to violaceous plaques with multilayered silvery scale, nail pitting, and oil-drop changes; PASI was 17.4 in 2021. He reported a history of arthralgia, but no joint swelling, deformity, or tenderness was documented on examination, and no diagnosis of psoriatic arthritis had been established. In October 2021, routine testing during dermatology care showed serum creatinine of 118 μmol/L, estimated glomerular filtration rate (eGFR) of 64.3 mL/min/1.73 m², hyperuricemia, asymptomatic proteinuria, and occult hematuria. Kidney biopsy was advised, but the patient declined. Between September 2022 and March 2024, serum creatinine increased from 166 to 377 μmol/L and eGFR declined from 42.5 to 15.6 mL/min/1.73 m².

At reassessment in March 2025, serum creatinine was 463 μmol/L, eGFR was 12.1 mL/min/1.73 m², and 24-hour urinary protein excretion was 4.70 g. At subsequent admission, the corresponding values were 407 μmol/L, 14.1 mL/min/1.73 m², and 2.90 g. Urinalysis showed an albumin-to-creatinine ratio of 359.36 mg/mmol, proteinuria (4+), and occult blood (2+). Autoimmune serologic testing was unremarkable. His medical history also included hypertension. Prior psoriasis treatments included secukinumab, apremilast, ixekizumab, and upadacitinib; however, no standardized renal response assessment was performed during those earlier treatment periods. Secukinumab had previously been stopped because of concern about infection risk. Upadacitinib was discontinued after the patient developed a left-eye central retinal vein occlusion requiring ophthalmologic treatment. Progressive kidney dysfunction and increased proteinuria had also been observed during this period, but a causal relationship between upadacitinib and the renal deterioration could not be established.

### Diagnostic assessment

**Figure 1:**
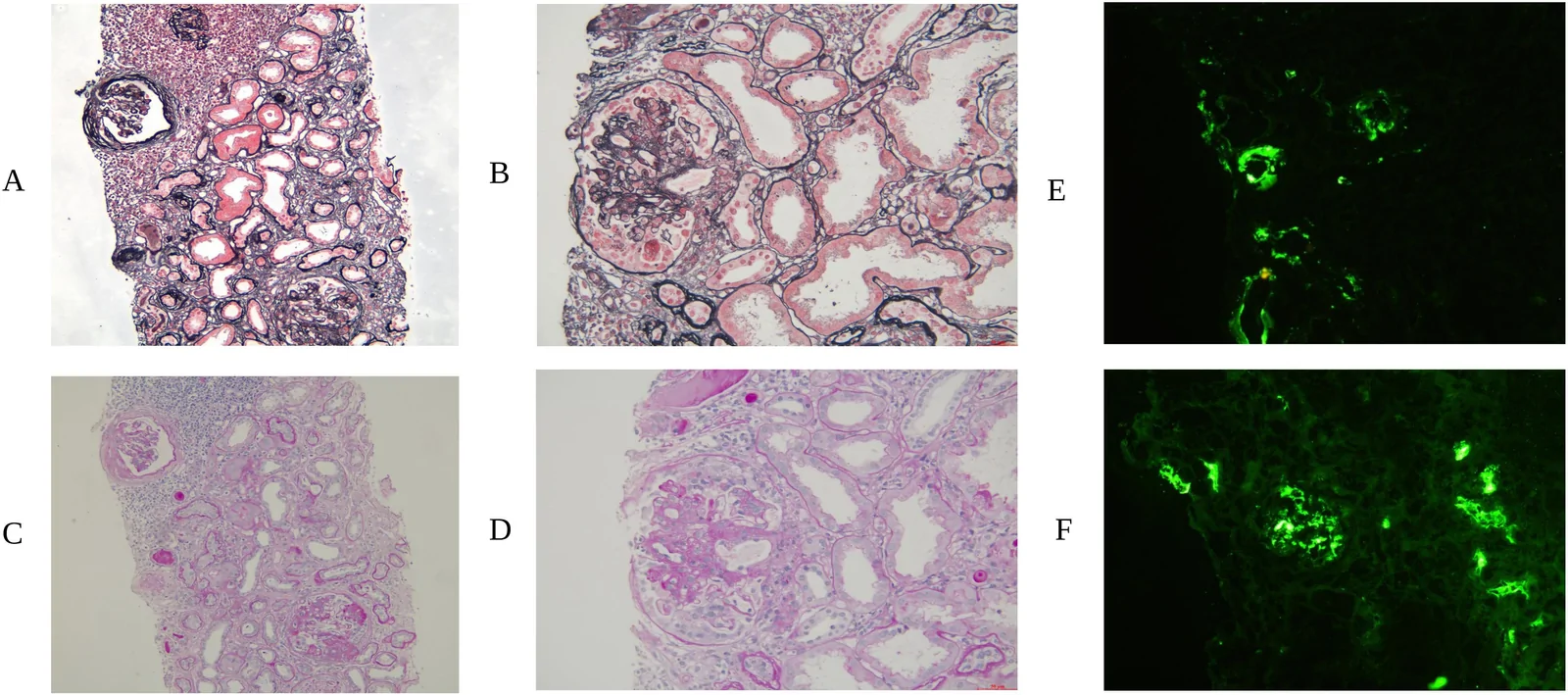
Representative kidney biopsy findings. **(A, B)** Periodic acid-methenamine silver staining showing ischemic glomerular sclerosis and chronic glomerular injury. **(C, D)** Periodic acid-Schiff staining showing mesangial hypercellularity and segmental sclerosis. **(E, F)** Immunofluorescence microscopy showing granular mesangial deposits of IgA (3+) and C3 (2+). Overall findings were consistent with proliferative sclerosing IgA nephropathy, Oxford classification M1E1S1T2C0, with ischemic injury and microangiopathy. Original magnifications: **(A, C)** ×100; **(B, D)** ×200.

Kidney biopsy showed eight glomeruli. One was globally sclerosed and four showed ischemic sclerosis. The remaining viable glomeruli showed diffuse mesangial hypercellularity, focal endocapillary hypercellularity, and focal segmental sclerosis. Tubulointerstitial lesions included tubular atrophy, mononuclear inflammatory infiltration, and fibrosis. Arteriolar changes consisted of luminal narrowing and segmental mucoid edema. Immunofluorescence microscopy showed granular mesangial IgA (3+) and C3 (2+) deposition, and immunostaining demonstrated granular C4d along glomerular capillary loops. The final pathologic diagnosis was proliferative sclerosing IgAN, Oxford classification M1E1S1T2C0, with ischemic glomerular injury and microangiopathy. Representative biopsy findings are shown in **Figure 1**.

The main diagnostic challenge was not the classification of IgAN, which was biopsy-proven, but the interpretation of disease activity and prognosis in a patient who already had advanced kidney dysfunction. The biopsy showed active mesangial and endocapillary lesions together with extensive chronic glomerular, tubulointerstitial, and vascular damage. This pattern made some short-term improvement biologically plausible, while making durable recovery of kidney function unlikely.

### Therapeutic intervention

Targeted-release budesonide 16 mg/day and deucravacitinib 6 mg/day were started in late March 2025. Although the PASI at initiation of the current treatment course was relatively low (2.4), the patient had a more than 10-year history of severe, relapsing plaque psoriasis, a previously documented PASI of 17.4, multiple prior systemic therapies, and persistent residual or recurrent lesions. Continued systemic treatment was therefore considered necessary by the dermatology team for ongoing disease control, and deucravacitinib was selected. BSA, sPGA, DLQI, and pruritus severity were not systematically recorded at treatment initiation. Background therapy consisted of nifedipine controlled-release 30 mg/day. No renin-angiotensin system blocker, sodium-glucose cotransporter 2 inhibitor, or mineralocorticoid receptor antagonist was used during the observed treatment period, and no major change in concomitant antihypertensive or dermatologic treatment occurred during the main observation window.

### Follow-up and outcomes

**Figure 2:**
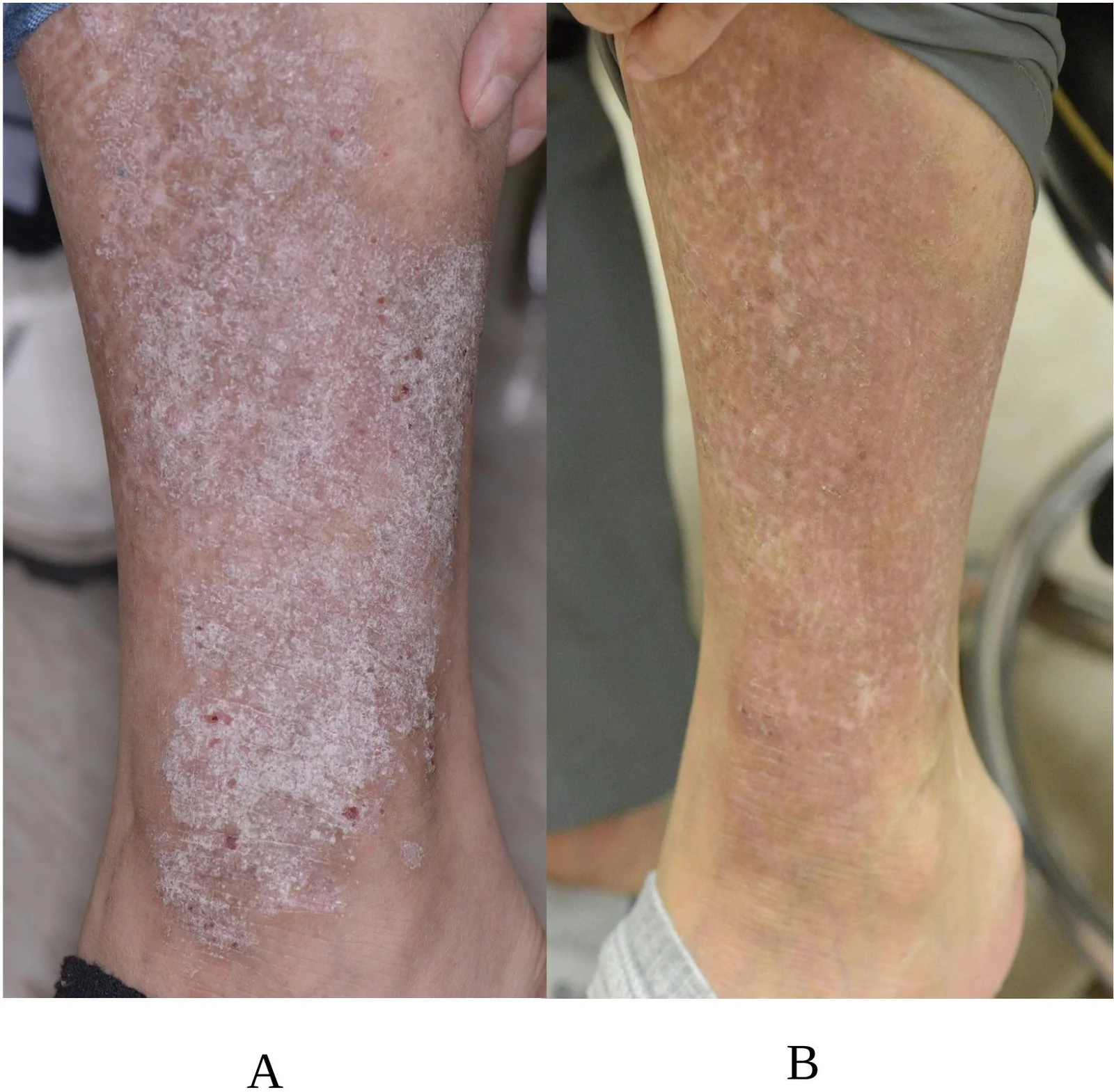
Psoriatic lesions before and after treatment. **(A)** Pretreatment photograph of the right lower extremity. **(B)** Photograph of the same anatomical site 3 months after treatment initiation. PASI decreased from 2.4 at baseline to 1.6 at follow-up, representing a modest change in cutaneous disease activity. Information on concomitant topical treatment during this interval was not systematically documented. Written consent for publication of the images was obtained.

During the initial treatment phase, 24-hour urinary protein excretion declined from a pretreatment range of 2.90-4.70 g to 0.62 g in August 2025. Serum creatinine decreased from 463 to 323 μmol/L, and eGFR increased from 12.1 to 18.6 mL/min/1.73 m². In September 2025, serum creatinine reached 278 μmol/L and eGFR reached 22.3 mL/min/1.73 m². A modest improvement in cutaneous disease activity was observed, with the Psoriasis Area and Severity Index decreasing from 2.4 to 1.6. Representative clinical photographs are shown in **Figure 2**.

The patient self-discontinued deucravacitinib in August 2025. Proteinuria increased from 0.62 g in August to 1.27 g in September and 4.70 g in October 2025. Deucravacitinib was restarted in October. Proteinuria subsequently declined to 4.28 g in November and 3.94 g in December 2025. Serum creatinine was 312, 335, and 357 μmol/L in October, November, and December, respectively, with corresponding eGFR values of 19.4, 17.8, and 16.5 mL/min/1.73 m2. Targeted-release budesonide was stopped in December 2025 after completion of the planned 9-month course.

**Figure 3:**
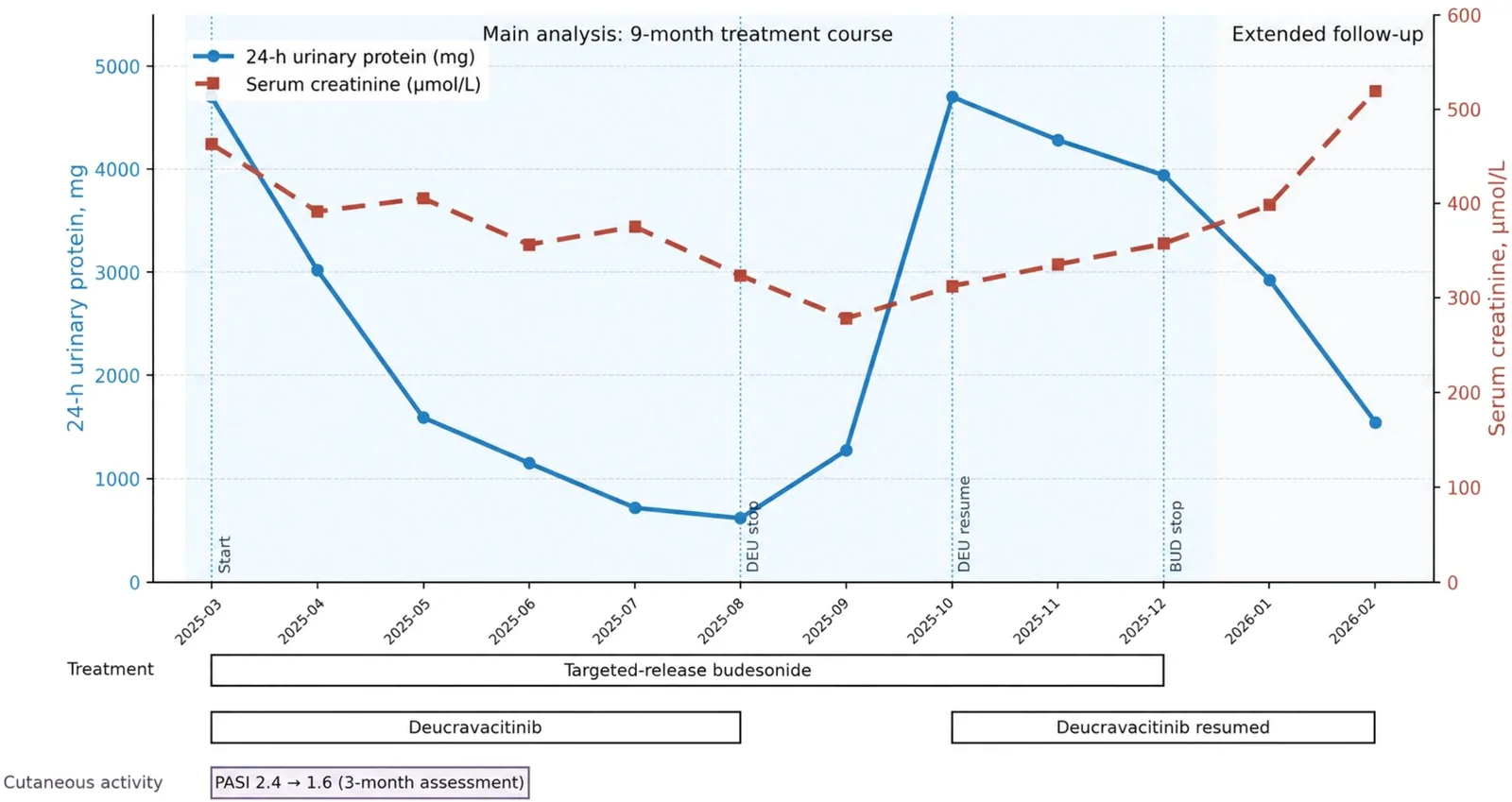
Clinical course and treatment timeline. Serial changes in 24-hour urinary protein excretion and serum creatinine from March 2025 to February 2026 are shown together with the two available PASI assessments (2.4 at treatment initiation and 1.6 at the 3-month dermatologic follow-up). Targeted-release budesonide (BUD) and deucravacitinib (DEU) were initiated in late March 2025. Deucravacitinib was self-discontinued in August 2025 and resumed in October 2025. Targeted-release budesonide was discontinued in December 2025 after completion of the planned treatment course. January and February 2026 are shown as extended follow-up.

During extended follow-up under deucravacitinib monotherapy, 24-hour urinary protein excretion declined to 2.92 g in January 2026 and 1.54 g in February 2026. In contrast, kidney function worsened: serum creatinine increased to 398 μmol/L in January and 519 μmol/L in February, while eGFR declined to 14.5 and 10.4 mL/min/1.73 m², respectively. The clinical timeline is summarized in **Figure 3**. During deucravacitinib treatment, no infection, including herpes zoster, oral ulceration, acneiform eruption, or folliculitis was observed. No clinically significant treatment-emergent abnormalities in liver enzymes, lipid parameters, or peripheral blood cell counts were documented during the observed treatment period, and no serious adverse event occurred.

### Timeline

The care timeline, including initiation and discontinuation of targeted-release budesonide and deucravacitinib, serial 24-hour urinary protein excretion and serum creatinine values, and the two available PASI assessments (2.4 at treatment initiation and 1.6 at the 3-month dermatologic follow-up), is presented in **Figure 3**.

## Discussion

This case is notable less for an apparent treatment response than for the way the biopsy constrained its interpretation. The specimen showed active mesangial and endocapillary lesions, but these coexisted with extensive chronic injury, including glomerulosclerosis, ischemic sclerosis, tubulointerstitial fibrosis, tubular atrophy, and vascular injury. This mixed pattern made partial improvement in proteinuria biologically plausible, while also making full recovery of kidney function unlikely. The later dissociation between falling proteinuria and worsening eGFR was therefore not paradoxical; it was compatible with advanced structural disease.

Previous reports have described IgAN in patients with psoriasis, sometimes under the debated concept of psoriatic nephropathy (3–8). Early clinical reports and small series described hematuria, proteinuria, nephrotic-range proteinuria, crescentic changes, vascular nephrosclerosis, and tubulointerstitial injury in psoriatic patients with biopsy-proven IgAN (5, 6). Later reports broadened the clinical spectrum to erythrodermic psoriasis and rapidly progressive or crescentic IgA-dominant glomerulonephritis (7, 8). A recent clinicopathologic study emphasized that psoriasis-associated IgAN remains insufficiently characterized, especially with regard to prognosis and whether its features simply mirror primary IgAN (4).

Treatment observations in the literature remain anecdotal. Selected cases have described improvement after control of psoriasis or IL-17A blockade, including secukinumab-based treatment, but these reports were limited by concomitant interventions, short follow-up, or single-patient design (9, 10). These observations are consistent with a clinical association and a biologically plausible but unproven inflammatory connection between psoriasis and IgAN, but they are not sufficient to define a shared disease mechanism or treatment pathway. The current case therefore should not be presented as a first-line therapeutic proposal. Its value lies in linking biopsy-defined activity and chronicity to a longitudinal course in which skin disease, proteinuria, and eGFR did not behave as a single unified outcome.

Targeted-release budesonide has a clear biologic rationale in IgAN because it is designed to act at mucosal lymphoid tissue in the distal ileum and reduce pathogenic immune activation relevant to galactose-deficient IgA1 production (11–13). In trials, this approach reduced proteinuria and attenuated eGFR decline in patients with IgAN, although patients with very advanced kidney dysfunction were generally under-represented (11, 12). More recent reports suggest that selected patients with severe renal impairment may still show short-term reductions in proteinuria (13). In this patient, the early response during budesonide and deucravacitinib combination therapy cannot be separated into drug-specific effects.

**Figure 4:**
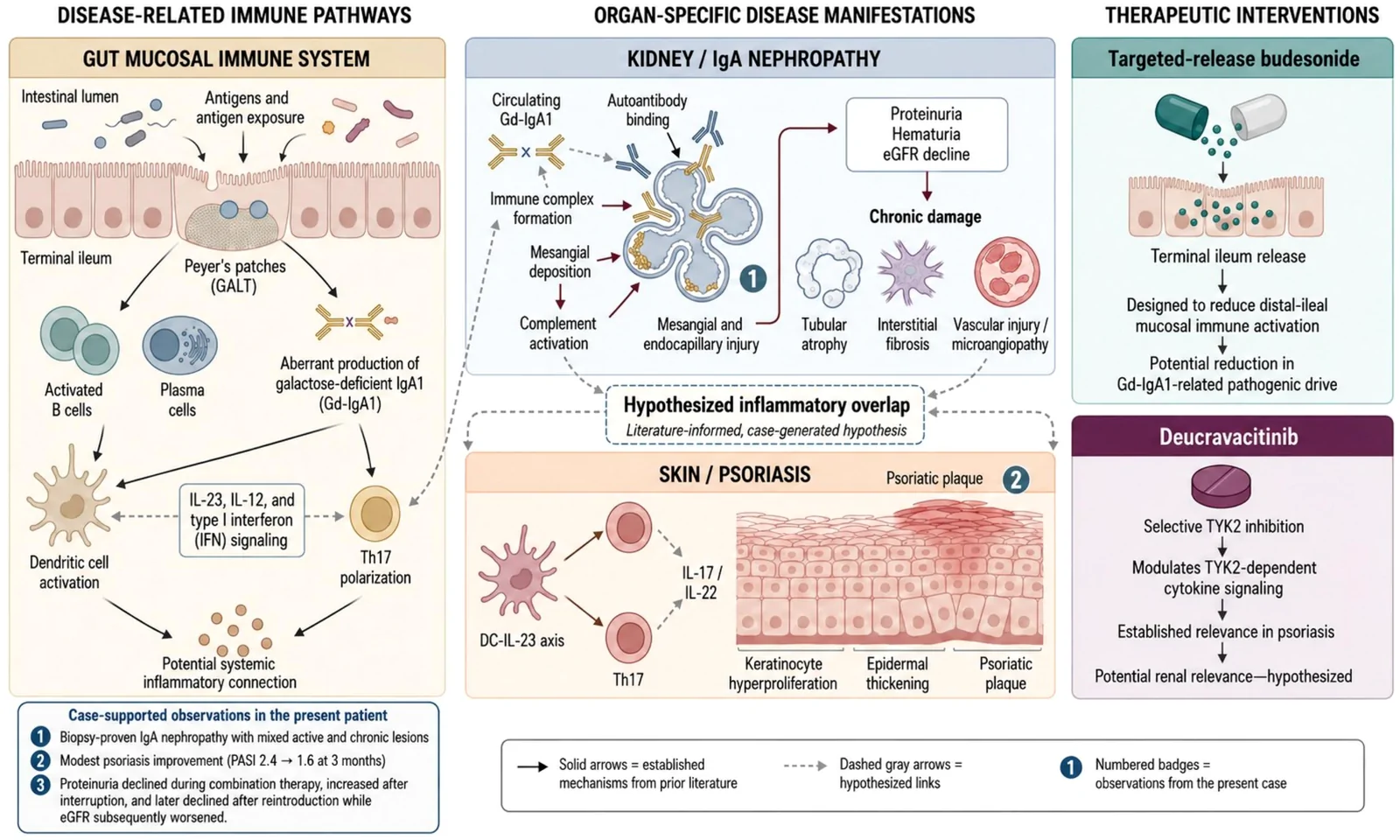
Literature-informed conceptual framework of psoriasis and IgA nephropathy with case-supported observations. Literature-supported components include mucosal immune dysregulation, galactose-deficient IgA1 production, immune-complex formation, and downstream renal injury in IgA nephropathy, as well as IL-23/TYK2/Th17 signaling in psoriasis. Observations from the present case include biopsy-proven IgAN with mixed active and chronic lesions, a modest reduction in PASI, changes in proteinuria, and later deterioration in kidney function. Solid arrows indicate literature-supported mechanisms, whereas dashed arrows indicate hypothesized, not established, inflammatory links between psoriasis and IgA nephropathy and a possible contribution of TYK2-pathway modulation. The figure is conceptual and does not represent direct measurement of TYK2-pathway activity, cytokines, complement activation, or galactose-deficient IgA1 in this patient.

Deucravacitinib is a selective allosteric TYK2 inhibitor with established efficacy in plaque psoriasis (14, 15). TYK2 mediates signaling downstream of IL-23, IL-12, and type I interferons. A biologically plausible but unproven inflammatory connection between psoriasis and IgAN may involve IL-23/TYK2/Th17 signaling, with additional contributions from mediators such as IL-6 and TNF-alpha (16–18). This literature-informed framework is summarized in **Figure 4**. In the present case, the fall in proteinuria after reintroduction of deucravacitinib and the subsequent decline during deucravacitinib monotherapy were compatible with, but did not establish, a possible contribution of TYK2 inhibition. The concurrent deterioration in eGFR argues against interpreting proteinuria improvement as reversal of advanced chronic kidney damage. Accordingly, the patient-level observations should be viewed as hypothesis-generating rather than as evidence of a shared inflammatory mechanism.

The strengths of this report include biopsy confirmation, detailed clinicopathologic description, serial laboratory follow-up, and documentation of both renal and cutaneous findings. The limitations are substantial. This was a single case. Two agents were started together, and the later period was not a clean rechallenge because budesonide was completed and kidney function was changing. The biopsy specimen was limited in size, although concordant glomerular, tubulointerstitial, and vascular chronic lesions supported the overall interpretation. Mechanistic interpretation was limited by the absence of serial disease-specific biomarkers, including galactose-deficient IgA1, complement activation markers, cytokine profiles, or direct measures of TYK2-pathway activity. Only two PASI assessments were available during the current treatment period, and BSA, sPGA, DLQI, and pruritus severity were not systematically recorded; therefore, the cutaneous response should not be overinterpreted. Prior secukinumab, apremilast, ixekizumab, and upadacitinib exposure was clinically relevant but could not be evaluated as a standardized renal treatment comparison. The absence of renin-angiotensin system blockade, sodium-glucose cotransporter 2 inhibition, and mineralocorticoid receptor antagonism during the observed period reduces some treatment-related confounding, but it also limits generalizability and should be considered when interpreting the proteinuria trajectory.

The main lesson is that kidney biopsy remains central when clinical signals are complex. In this patient, pathology established the diagnosis, defined the active and chronic components of injury, and helped explain why proteinuria and kidney function later moved in different directions. The longitudinal course, together with the known clinical association between psoriasis and IgAN, generates a testable hypothesis of a possible inflammatory connection between the two diseases; however, it should not be taken as evidence that a shared inflammatory mechanism is established or that TYK2 inhibition is effective for IgAN.

### Patient perspective

The patient reported that the improvement in urinary foam and skin lesions during the initial treatment period was encouraging. He understood that improvement in proteinuria did not necessarily indicate reversal of chronic kidney damage and agreed to continued nephrology and dermatology follow-up.

## Data Availability

The original contributions presented in the study are included in the article/supplementary material. Further inquiries can be directed to the corresponding author.
